# Hope level and associated factors among parents of retinoblastoma patients during COVID-19 pandemic: a cross-sectional study

**DOI:** 10.1186/s12888-021-03401-0

**Published:** 2021-08-06

**Authors:** Changjuan Zeng, Wenting Cao, Ting Zhao, Li Li, Lili Hou

**Affiliations:** 1 Department of Nursing, Ninth People’s Hospital, Shanghai Jiao Tong University School of Medicine, Shanghai, China; 2 Department of Ophthalmology, Ninth People’s Hospital, Shanghai Jiao Tong University School of Medicine, Shanghai, China; 3 Shanghai Key Laboratory of Orbital Diseases and Ocular Oncology, Shanghai, China; 4 Shanghai JiaoTong University School of Nursing, Shanghai, China; 5 School of Nursing and Health Management, Shanghai University of Medicine & Health Sciences, Shanghai, China

**Keywords:** Hope level, Depression, Anxiety, Risk factors, Retinoblastoma, Pediatric oncology, COVID-19

## Abstract

**Background:**

The 2019 coronavirus disease (COVID-19) is a global public health emergency. So far, only a limited number of studies have investigated the impact of the COVID-19 pneumonia pandemic on the mental health of parents having children with cancer. This study investigated the hope, and relevant influencing factors (depression, anxiety, demographic data) among parents whose children had retinoblastoma (RB) during the COVID-19 pandemic.

**Methods:**

A cross-sectional survey and a convenient sampling survey were conducted on 317 parents of RB children admitted to the Ninth People’s Hospital affiliated with Shanghai Jiao Tong University, School of Medicine, and Qilu Children’s Hospital of Shandong University. The survey tools included Demographic Questionnaire, Herth Hope Index (HHI), The Generalized Anxiety Disorder (GAD-7), and the Patient Health Questionnaires (PHQ)-2.

**Results:**

The hope level score of the parents of RB patients was (35.36 ± 4.42), which was at the medium level. The highest dimension of hope score was inner positive readiness and expectancy (12.07 ± 1.57), and the lowest dimension was interconnectedness with self and others (11.50 ± 1.64). The incidence rate of depression and anxiety was 29.97% (95/317) and 41.32% (131/317), respectively. Monoculus or binoculus disease, sleep status, health status, and depression /anxiety had statistically significant effects on the parental hope level (*p* < 0.05). Multiple linear regression analysis revealed that time since diagnosis, education level, treatment type and depression were independent influencing factors (*p* < 0.05), accounting for 22.60% of the variation in hope level.

**Conclusion:**

During the COVID-19 pandemic, medical staff should formulate targeted intervention measures according to different characteristics of ocular disease, time since diagnosis, treatment type, parental educational level and emotional state in order to improve the level of parental hope.

## Background

In 2019, a novel COVID-19 pneumonia epidemic first broke out in Wuhan City, Hubei Province, China, and it quickly spread worldwide, becoming a global public health emergency according to the World Health Organization (WHO) [1, 2]. This new type of coronavirus is highly contagious. It spreads fast and can infect people of all ages [3]. The transmission of the virus reduced the mental health and increased depression and anxiety of the public. In addition, the sleep quality for many people has deteriorated [4, 5]. The WHO advocates social distancing and self-isolation [6]. Many provinces in China have taken measures to restrict travel and personnel mobility, which have affected the treatment and re-examination of cancer patients to a certain extent. Family caregivers of cancer patients face unprecedented uncertainty and are prone to depression, especially under the double threats of loneliness and COVID-19 [7]. 17.7% of informal caregivers (58.9% of spouses) of cancer patients over 18 years old have been reported to have suicidal ideation, while 2.8% have attempted suicide during the past year [8]. Moreover, elderly spousal caregivers who reported caregiving-related strain had a 63% higher four-year all-cause mortality rate than non-caregiving controls [9]. Parents of children with cancer are often confronted with uncertainty, anxiety, stress, and coping with feelings out of control [10]. Family caregivers of patients need to be concerned, especially the parents whose children had tumors.

The role of hope is a hot topic in oncology care and treatment. Hope is defined as a multidimensional and broad concept that is important to all cancer patients’ parents [11, 12]. As the fundamental source of inner strength and psychological power, hope is an important coping skill that drives parents to provide patients with continuous care and support [13]. Hope is a positive driving force that influences a person’s behavior and manner. It can also improve confidence and courage [14]. The hope of parents in caregiving may greatly improve children’s quality of life [15], treatment effect [16], and alleviate parental psychological pain and poor adaptation after a cancer diagnosis of their children [17].

However, existing research on parental hope in the context of children’s disease experience is limited [12]. There are few reports on parental hope in children’s cancer or have focused on advanced cancer and poor prognosis [13]. Hope is considered as a critical perception in nursing. When caring for children suffering from cancer and facilitating their parents, the most important thing is to minimize the factors that harm the parents’ hopes and cultivate the factors that enhance their hopes [12]. RB is the most common primary intraocular malignant tumor in children, which leads to decreased visual function and can be life-threatening [18]. It also brings economic burden and mental stress to society and the whole family [19, 20]. This study aimed to investigate the hope, depression, anxiety among parents of RB patients during the COVID-19 epidemic, and to explore the influencing factors. The findings may provide theoretical support for the development of applicable intervention programs in the future to improve the hope level of parents whose children had RB tumors.

## Methods

### Participants

A cross-sectional survey and a convenient sampling survey were adopted. Parents of RB patients (only the father or mother of each child’s family was investigated) were recruited as research subjects. Inclusion criteria were following: (a) the participating parent is the primary caregiver of the child(i.e. who lived with and took more than 6 months care of the RB child); (b) volunteered to participate in the survey; (c) over 18 years of age; (d) with no cognitive impairment (such as delusions or dementia) or other serious illness that could lead to inability to participate in this survey independently; (e) with no other major family life threatening events occurring recently (such as death of family members). Exclusion criteria were: (a) whose child with advanced RB (such as brain metastasis or systemic metastasis) and/or were receiving palliative care; (b) whose child with other serious diseases (such as severe heart disease or other tumors).

### Data collection

Our research team used a WeChat platform specifically for the out-of-hospital management of all RB children treated in the Ninth People’s Hospital, Shanghai Jiao Tong University School of Medicine and Qilu Children’s Hospital of Shandong University. On March 9th, 2020, we published an advertisement via WeChat platform to introduce the purpose, significance and research process of this survey. Then, according to the inclusion and exclusion criteria, the eligible parents of RB children were selected to participate in our study. For the selected participants, we released an online questionnaire (https://www.wjx.cn) via WeChat platform. The introduction part of the questionnaire explained the ways to fill out the survey. All subjects who were willing to participate in the survey filled out and submitted the questionnaire online. The survey was confidential and anonymous. The recruitment process continued until June 1st, 2020. A total of 320 subjects were invited to participate in the study, and 3 subjects refused. Finally, 317 valid questionnaires were collected, and the effective rate was 99.06%.

### Questionnaires

#### Demographic questionnaire

This self-report questionnaire designed by the authors included the age of the patient’s father or mother, place of residence, marital status, education, occupation, sleep status, health status, family income, knowledge of RB (nothing at all, know a little, know), patient’s age, sex, time since diagnosis, ocular disease (monoculus or binoculus), times of hospitalizations, disease status (under treatment, under re-examination, cured), tumor stage at diagnosis (including [21] extraocular RB and intraocular RB,classified by the International Classification of RB), treatment types. The variable ‘sleep status’, ‘health status’ were rated on a five-point scale ranging from “very good” =1 to “very poor” =5. The parent’s age and the patient’ age, time since diagnosis, treatment types, times of hospitalizations are divided into different levels.

#### Herth Hope index (HHI)

The scale was originally designed by Herth et al. The internal consistency was 0.89 to 0.97, and the test-retest reliability coefficient was 0.91 for a variety of adult patient populations [22]. We used the Chinese version edited by Zhao and Wang Jian (scoring range12–48), which was a self-report scale consisting of 12 items (including 4 reaction categories: 1 = strongly disagree to 4 = strongly agree). The level of hope was evaluated from 3 dimensions comprising of inner sense of temporality and future, inner positive readiness and expectancy and interconnectedness with self and others. Scores were divided into 0 level (low) (12 < HHI < 23), 1 level (medium) (24 < HHI < 35), and 2 level (high) (36 < HHI < 48) [23]. A higher HHI score indicated a higher level of hope [22]. The Herth Hope Scale has shown satisfactory reliability and validity in different Chinese samples [24, 25], family caregivers of patients with cancer [26], and the general population [27].

#### Patient health Questionnaires-2 (PHQ-2)

PHQ-2, extracted from 9 items of the Patient Health Questionnaire (PHQ-9), was used to investigate the symptoms of depression. PHQ − 2 is a 4-point Likert scale, ranging from 0 (not at all) to 3 (almost every day) [28]. The total score ranges from zero to six points. It evaluates the frequency of “feeling down, depressed or hopeless” and “little interest or pleasure in doing things” experienced over the past 2 weeks. The sensitivity and specificity for the diagnosis of depression with a PHQ-2 score of 2 or higher were 86 and 78%, respectively, and 61 and 92% with a score of 3 or higher [29]. A study in China showed that the sensitivity of screening with a cut-off value of PHQ-2 score of 3 points was further improved compared to 2 points [30]. Therefore, in our study, a score of 3 points was used as the screening value. Values from 0 to 2 points were considered as normal, and 3 to 6 points were considered suspicious. We further classified the suspicious groups into parents with depression and without depression. PHQ-2 has been used and validated in China [31, 32].

#### The generalized anxiety disorder (GAD-7)

The GAD-7, developed by Spiter et al., is a practical self-report anxiety questionnaire to screen for generalized anxiety and assess symptom severity. The GAD-7 is a response to the patient’s mental psychological activity over the past 2 weeks. It contains 7 entries, with each entry question corresponding to 4 options. GAD-7 uses a 4-point scale from 0 to 3, where 0 = no at all, 1 = several days, 2 = more than half of the days, and 3 = nearly every day. The total score is 0 to 21points, with higher scores indicating more severe symptoms. A total score equal to or greater than 10 is used as the cut-off value for screening GAD [33]. Values from 0 to 21 were considered as normal, and 10 to 21 points were suspicious ones. The GAD-7 has been shown to be effective in primary care [34, 35]. It application in Chinese population has also been proven to have good reliability and validity [36, 37].

### Statistical analysis

SPSS (version.25), R software (version 3.6.1) and various R packages were used for statistical analyses. Descriptive statistics were used to describe the demographic and the clinical characteristics of samples. Continuous variables were expressed with mean ± standard deviation. Categorical variables were represented with number and percentage (n, %).

Univariate analysis was used to assess the association between hope and independent variables. Independent sample t-test was used to determine the difference of hope level between two groups. One-way analysis of variance (ANOVA) was used to determine the difference of hope level among three or more independent groups. Multivariate linear regression analysis was used to determine the predictors of hope. After univariate analysis, the variables with *P* values less than 0.2 were included in the multivariate linear regression equation. The model with the lowest Akaike Information Criterion (AIC) value was selected as the best-fitting model. Multivariate linear regression was performed in R using the lm () function, and stepwise regression was performed in R using the step AIC () function. The stepwise regression method used AIC as the criterion to calculate the AIC values of different regression equations (deleted and added variables). The regression equation with the smallest AIC value was selected as the optimal regression equation. Statistical significance was set at a *P* value < 0.05.

### Ethical considerations

The research was reviewed by the Institutional Review Board in the researcher’s hospital, which was in line with the Declaration of Helsinki [38]. All selected subjects were informed of the purpose and nature of the study, and were told that they could withdraw from the study at any time, and were promised that their personal information would not be disclosed.

## Results

### Sample characteristics

The demographic characteristics of parents and RB patients was presented in Table 1 and Table 2, respectively. The parent participants consisted of both mother (64.67%) and father (35.33%), and the ratio of mother to father was approximately 2:1. Most of the participants were over 29 years old (66.25%), married (97.16%), with an education level above elementary school (95.90%), living in countryside (38.80%), having general sleep status (63.09%) and good health status (43.85%), working (58.99%), with a family income that could not meet children’s medical expenses (53.00%), and having an understanding of RB (93.69%). Among the 317 parents who were enrolled in the assessment, 95(29.97%)parents were screened suspicious for depression at the cut-off point of 3. Additionally, 131 (41.32%) parents were screened suspicious for anxiety at the cut-off point of 10. The age of 317 RB patients ranged from 1 month to 172 months, with an average age of 41.38 ± 23.14 months, and 84.22% were younger than 5 years old. 65.62% of the patients had been diagnosed with RB for more than 1 year. The ratio of male to female was about 1:1. Most of the patients had monoculus (70.98%), received 4–6 types of treatment (49.84%), had more than 10 hospitalizations (84.22%), and were under re-examination (64.98%). Over 90% of the RB cases were classified into D stage (41.64%) or E stage (49.21%). Univariate analysis showed significant differences in the scores of hope level among the subjects with different ocular disease s, sleep status, health status, depression and anxiety (all *P*<0.05). Despite the statistical significance, it remained unclear whether these quite small differences in HHI were clinically meaningful.

**Table 1:** Demographic characteristics of parents with different hope scores (*N* = 317)

Characteristics	N (%)	hope score(^**−**^x ±s)	F-value /t-value	***P***-value
**Relationship to patient**				
Mother	205(64.67)	35.17 ± 4.44	−1.056^#^	0.292^a^
Father	112(35.33)	35.71 ± 4.39		
**Age, years**				
≤ 29	107(33.75)	34.64 ± 4.33	2.129^*^	0.121
29–35	134(42.27)	35.74 ± 4.66		
> 35	76(23.98)	35.70 ± 4.03		
**Residence**				
City	115(36.28)	36.08 ± 4.17	2.486^*^	0.085
Town	79(24.92)	34.80 ± 4.98		
Countryside	123(38.80)	35.05 ± 4.20		
**Marital status**				
Married	308(97.16)	35.41 ± 4.44	1.309^*^	0.272
Divorced	6(1.89)	34.83 ± 3.71		
Separated	3(0.95)	31.33 ± 2.52		
**Education**				
Less than Primary	13(4.10)	35.38 ± 4.82	1.656^*^	0.160
Junior high school	101(31.86)	34.48 ± 4.17		
High school or technical secondary school	80(25.24)	35.85 ± 4.25		
College or undergraduate	114(35.96)	35.68 ± 4.66		
Postgraduate	9(2.84)	36.78 ± 4.24		
**Occupation**				
Job	187(58.99)	35.64 ± 4.48	−1.365^#^	.173 ^a^
Jobless	130(41.01)	34.95 ± 4.32		
**Sleep status**				
Good	61(19.24)	36.82 ± 5.06	4.426^*^	**0.005**
General	200(63.09)	35.30 ± 3.89		
Poor	22(6.94)	34.68 ± 5.70		
Very poor	34(10.73)	33.56 ± 4.61		
**Health status**				
Very good	64(20.19)	36.81 ± 4.46	3.637^*^	**0.013**
Good	139(43.85)	35.35 ± 4.42		
General	107(33.75)	34.55 ± 4.14		
Poor	7(2.21)	34.71 ± 5.82		
**Can family income meet children’s medical expenses**				
Can	149(47.00)	35.79 ± 4.35	1.645^#^	0.101
Can’t	168(53.00)	34.98 ± 4.46		
**Understanding of RB**				
Not at all	20(6.31)	35.05 ± 5.45	0.405^*^	0.667
Know a little	262(82.65)	35.30 ± 4.26		
Know	35(11.04)	35.97 ± 5.00		
**Depression**				
PHQ ≥ 3	95(29.97)	32.59 ± 4.05	7.993^#^	**< 0.001**^a^
PHQ < 3	222(70.03)	36.55 ± 4.03		
**Anxiety**				
GAD≥10	131(41.32)	33.79 ± 4.55	5.566^#^	**< 0.001**^a^
GAD < 10	186(58.68)	36.47 ± 3.98		

*Note*. PHQ The patient health questionnaire; GAD generalized anxiety disorder; N Number; * = F value; # = t value; ^a^ *P*-values calculated from Independent sample t-test; all other *P*-values were obtained by using the One-way ANOVA test; Bold face *p* < 0.05.

**Table 2:** Demographic characteristics of RB patients with different hope scores (N = 317)

Characteristics	N (%)	hope score $\left(\overline{\boldsymbol{x}}\pm \boldsymbol{s}\right)$	F-value /t-value	***P***-value
**Age, years**				
≤ 2	60(18.93)	35.82 ± 4.15	0.337^*^	0.799
> 2,≤3	90(28.39)	35.09 ± 3.57		
> 3,≤5	117(35.91)	35.30 ± 4.85		
> 5	50(15.78)	35.44 ± 5.10		
**Sex**				
Male	167(52.68)	35.06 ± 4.94	−1.294^#^	0.197^C^
Female	150(47.32)	35.69 ± 3.74		
**Time since diagnosis, months**				
≤ 6	44(13.88)	36.02 ± 4.72	0.930^*^	0.447
> 6,≤12	65(20.50)	35.09 ± 4.06		
> 12,≤24	102(32.18)	35.65 ± 4.29		
> 24,≤36	75(23.66)	35.28 ± 4.40		
> 36	31(9.78)	34.23 ± 5.14		
**Ocular disease**				
Monoculus	225(70.98)	35.52 ± 4.27	−2.303^#^	**0.022**^C^
Binoculus	92(29.02)	34.97 ± 4.76		
**Tumor stage at diagnosis**^**a**^				
A/B/C	15(4.73)	35.80 ± 3.12	0.580^*^	0.629
D	132(41.64)	35.43 ± 4.38		
E	156(49.21)	35.14 ± 4.55		
Extraocular	14(4.42)	36.64 ± 4.60		
**Treatment types** ^**b**^				
≤ 4	131(41.32)	34.90 ± 4.61	2.972^*^	0.053
4–6	158(49.84)	35.94 ± 4.36		
> 6	28(8.84)	34.25 ± 3.42		
**Times of hospitalizations**				
≤ 6	110(34.70)	35.36 ± 4.26	1.650^*^	0.194
6–10	94(29.65)	35.97 ± 4.31		
> 10	113(35.65)	34.85 ± 4.64		
**Disease state**				
Under treatment	108(34.07)	34.72 ± 4.09	1.711^*^	0.182
Under reexamine	206(64.98)	35.69 ± 4.59		
Had cured	3(0.95)	35.60 ± 7.58		

*Note*. N Number; * = F value; # = t value; Bold face *p* < 0.05.

^a^intraocular retinoblastoma was classified by the International Classification of Retinoblastoma as group A; group B; group C; group D; and group E.

^b^the sum of the treatment types methods (such as intravenous chemotherapy, intra-arterial chemotherapy, examination under anesthesia, enucleation, intracameral chemotherapy, intravitreal chemotherapy, cataract surgery, onsolidation therapies, pars plana vitrectomy, anti-neovasculariztion therapy, and cradiation-based therapies) that have been used so far.

^C^*P-*values calculated from Independent sample t-test; all other *P-*values were obtained by using the One-way ANOVA test.

### Data of hope level

The total score of hope level in parents of patients with RB was 35.36 ± 4.42. Scores of each level and dimension were listed in Table 3 and Table 4. Among them, 3 cases (0.95%) had a low hope level, 155 cases (48.90%) had a medium hope level, and 159 cases (50.15%) had a high hope level. The highest scored hope dimension was inner positive readiness and expectancy, while the lowest scored dimension was interconnectedness with self and others.

**Table 3:** Distribution of hope level in each dimension (N = 317)

Hope level	N (%)	Inner sense of temporality and future $\left(\overline{\boldsymbol{x}}\pm \boldsymbol{s}\right)$	Inner positive readiness and expectancy $\left(\overline{\boldsymbol{x}}\pm \boldsymbol{s}\right)$	Interconnectedness with self and others $\left(\overline{\boldsymbol{x}}\pm \boldsymbol{s}\right)$
**low** (12 < HHI < 23)	3(0.95)	7.33 ± 0.58	5.00 ± 1.00	6.67 ± 0.58
**Medium** (24 < HHI < 35)	155(48.90)	10.74 ± 1.12	11.34 ± 0.96	10.48 ± 1.11
**High** (36 < HHI < 48)	159(50.15)	12.89 ± 1.18	12.92 ± 1.36	12.58 ± 1.23

**Table 4:** The hope score ranking in each dimension (N = 317)

Dimensions	score(^**−**^x ±s)	score sort
**Inner sense of temporality and future**	11.79 ± 1.63	2
**Inner positive readiness and expectancy**	12.07 ± 1.57	1
**Interconnectedness with self and others**	11.50 ± 1.64	3
**Total score**	35.36 ± 4.42	

### Multivariate linear regression analysis of hope

The results of a multiple regression analysis with the level of hope as the dependent variable was shown in Table 5. Multivariate linear regression analysis revealed that education had a significant positive correlation with hope level (β = 0.893, *p* < 0.001), whereas there was a significant negative correlation of hope level with time since diagnosis (β = − 0.489, *p* < 0.05), treatment types (β = − 0.958, *p* < 0.05) and depression (β = − 3.393, *p* < 0.001). Adjusted R square (R^2^) of the model was 0.232, which indicated that this model accounted for 23.20% of the variance of the dependent variable.

**Table 5:** Multivariate linear regression analysis of hope among parents of RB patients

Predictor	β	SE	t	***P***
Intercept	34.342	1.4	24.527	**< 0.001**
Time since diagnosis	−0.489	0.24	−2.036	**< 0.05**
Treatment types	−0.958	0.411	−2.329	**< 0.05**
Times of hospitalizations	0.528	0.34	1.554	0.121
Parents’ age	0.425	0.297	1.465	0.144
Education	0.893	0.228	3.923	**< 0.001**
Sleep status	−0.42	0.276	−1.523	0.129
Depression	−3.693	0.484	−7.623	**< 0.001**
Disease state	0.88	0.507	1.734	0.084

*Note*. The coefficient (β) in the model indicates the correlation (positive or negative) and the significance of the relationship to the outcome variable (hope). Residual standard error: 3.872 on 308 degrees of freedom. Multiple R-squared: 0.2519, Adjusted R-squared: 0.2324,F-statistic: 12.96 on 8 and 308 DF, *p-*value< 0.001, AIC (Akaike Information Criterion): 867.2

## Discussion

### Distribution of parental hope level

This study showed that during the COVID-19 pandemic, 48.90% (155/317) parents of RB patients had a middle level of hope, while 0.95% parents had a low level of hope. In contrast, Sisk et al [13] investigated the hope level of parents of pediatric cancer patients (183 cases of hematologic malignancy, 141 cases of solid tumor and 50 cases of brain tumor) in two academic pediatric hospitals in Boston and Philadelphia. They found that 55% of parents (206/374) had high levels of hope. The reason for this inconsistency may be due to the difference in survey time and survey subjects. Our survey was conducted during the COVID-19 outbreak, and the selected subjects were all from well-known tertiary general hospitals in China. Second, the health condition of RB children was not poor. In this study, 65.93% of the patients were simply re-examined or cured, and 95.58% of the patients were in the intraocular phase. The protection of eyes and eyesight was promising in most patients. Patients with unilateral RB accounted for 70.98%. A previous study reported that parents who adapted to RB diagnosis had a higher level of hope compared to those who had difficulty in adopting RB diagnosis [39]. In this survey, 65.62% of patients suffered with RB for more than 1 years, and their parents adapted to RB diagnosis. It was reported that parents defined hope as their understanding of the child’s health [40]. In this study, parents with an understanding of RB accounted for 93.69%. Therefore, most of the subjects received systematic health education and rehabilitation guidance, which was helpful to establish a correct understanding of rehabilitation and enhance their confidence and belief in rehabilitation. In addition, 97.16% of the subjects were married, while only 9 got divorced or separated. Married couples were likely to cope with difficulties more easily than divorced ones [41].

The highest scored dimension of the hope scale was inner positive readiness and expectancy, which indicated that the subjects had a positive attitude towards the rehabilitation of the patients, they were willing to take active action and were actively cooperating with doctors. The interconnectedness with self and others was the lowest scored dimension, probably because the respondents needed to spend a lot of time taking care of their children [17, 19], which affected their close relationship with others. In addition, during the investigation, the awareness of COVID-19 was increasing, and the social contacts were reduced [7]. This suggested that attention should be paid to the subjects’ social interaction in clinical caring care. Medical staff should encourage the participant to communicate with other family members and friends through online platforms such as messages and WeChat to keep hope and optimize happiness. The more positive attitude towards the disease, the subjects were more willing to take positive actions to improve the present situation. Communication with doctors and other health care professionals could significantly increase hope levels [42]. Even if the child’s prognosis is poor, the doctor’s disclosure of prognosis can bring hope [43]. Medical staff should be encouraged to communicate with the parents to understand the treatment, caring and prognosis of RB, so as to inspire realistic hope.

### Multivariate linear regression analysis results

Parental hope is a widespread multidimensional phenomenon, and the factors hindering and promoting hope levels are related to the parent him- or herself, other people, economics, faith, family pets, the adolescent, the care receiver and the care-giving personnel, the adolescent’s cancer and health status, and continuation of life [12]. The results of this study were slightly different, our findings showed that education, time since diagnosis, treatment types, and depression were the independent influencing factors (p<0.05), which could explain the 23.20% of the variation in the hope level.

### Protective factors of parental hope

The survey results showed that education level was a protective factor for hope level, which was consistent with the conclusions by Xiao et al [44] and Li et al [45]. People with high educational levels are more likely to obtain relevant knowledge and social resources on diseases and health, prepare psychologically and are less likely to develop negative emotions such as depression and anxiety. Higher education levels are associated with better adaptability, lower stress levels, less depression and anxiety, and active problem-solving abilities [42]. This also suggested that the government, the media, and medical staff should pay more attention to these groups and provide them with additional psychological support and help. For parents with low educational levels, clinical medical staff should be patient when giving education and using easy-to-understand words to guide them to cooperate and participate in treating children’s diseases actively.

### Risk factors of parental hope

The survey results showed that disease course and treatment types were negative factors affecting the hope level of parents having RB children, which was consistent with the findings by Xiao et al [44]. The longer course of the disease was associated with longer care time. The characteristics of caring work also make caregivers stay in a state under high pressure and high load for a long time, which may easily lead to a negative attitude of caregiver themselves during the rehabilitation treatment. In clinical work, medical staff should pay attention to assess the hope level of parents whose children had long-term RB, explain the illness, give information support, correct the understanding of the relationship between treatment and disease, reduce their uncertainty and helplessness, and enhance the hope in effectiveness of the treatment for patients.

The survey showed that the incidence rate of depression and anxiety among parents of children with RB was 29.97% (95/317) and 41.32% (131/317), respectively, which were higher than those of the general population (17.7 and 30.7%) [46]. This difference may be related to the variations in subject populations and assessment tools. Such a high incidence might be due to the outbreak of COVID-19. Tumor patients are susceptible to COVID-19 because of their low immunity when they go to the hospital. The lack of necessities caused by the pandemic has led to a decline in the nutritional status of cancer patients. The suspension of production during the pandemic further increased the economic burden of cancer patients’ families. Delaying or even stopping the treatment of a tumor may lead to the tumor’s progression or deterioration. Continuous assessment of spiritual needs is very important for keeping hope [14]. During the COVID-19 outbreak, 60% of caregivers with depressive symptoms complained of lacking treatment support (e.g., online or face to face) [7]. Better psychosocial and spiritual support could promote higher levels of hope [47]. It is suggested that the public health department, psychiatry department, health care department, nursing professionals, and family members of cancer patients should establish a cooperative partnership to ensure continuous care during the unique pneumonia outbreak of COVID-19. For people with depressive symptoms, necessary psychological counseling and intervention should be promptly conducted. Online platforms such as WeChat were used to popularize science propaganda on novel coronavirus and treatment or rehabilitation of tumor diseases to reduce patients anxiety.

### Clinical implications

This study helped health care professionals develop intervention measures for the mental health of parents of RB patients during the COVID-19 pandemic. When caring for cancer children and their parents, it is especially important to eliminate the factors that endanger the hopes of parents and foster the factors that generate hopes. These factors have been explored in this study. When communicating with parents of children with a long course of disease, low education level and a variety of treatment methods, easy-to-understand language should be used. In addition, the online clinics of oncology and psychiatry should be improved and expanded during the COVID-19 pandemic.

### Limitations

This study had a few limitations. First, the convenient sampling method was adopted in this study. The research samples came from two research centers, and the inclusion of parents with WeChat contact information could cause selection bias, which might affect the representation of the results to a certain extent. Future research could be conducted in multi-centers to generalize the results. Second, the parental hope changed over time [48], with different levels in different periods. However, this survey was a cross-sectional survey, and a longitudinal follow-up survey was not planned. In the follow-up studies, attention should be paid to the use of longitudinal research to understand the process of dynamic changes in hope. Third, the self-reported tools were used in this study, which was filled by the subjects themselves. It was inevitable that there would be some people’s misunderstanding of the question item and recall error might appear which might affect the results to some extent. In addition, we used GAD-7 and PHQ-2 as screening tools for anxiety and depression. Although some studies have reported that the two questionnaires have high reliability and validity [49], certain information might be omitted due to the limitation of items [29]. The suggested follow-up study can use other questionnaires to compare with the present findings. Furthermore, it was still not clear whether the pandemic had an influence on hope level and the predictors since no comparison to pre-pandemic states was made.

## Conclusions

The present survey investigated the impact of the novel coronavirus epidemic on families of RB patients, and showed that the hope level of patient’s parents was in the medium range, while the parents experienced different degrees of depression and anxiety. Hope level, which needed to be improved, was related to negative emotions. There was an urgent need for the implementation of hope-related assessments and intervention. However, this study showed that the time since diagnosis, education level, treatment types, and depression were particularly significant in predicting the level of hope. The society and individuals should make joint efforts to provide parents of RB patients with knowledge, attitude, and behavior intervention, and guide them in improving their hope level to win the psychological battle under the novel coronavirus pandemic.

## Data Availability

Data can be obtained from the corresponding author, on request.

## Abbreviations

COVID-19: The 2019 coronavirus disease
RB: Retinoblastoma
HHI: Herth Hope Index
GAD: The Generalized Anxiety Disorder
PHQ: The Patient Health Questionnaires
WHO: the World Health Organization
